# Assessing the effect of race on machine learning models predicting hospital admissions from emergency department

**DOI:** 10.1093/jamiaopen/ooag157

**Published:** 2026-08-10

**Authors:** Aidan Licoppe, Lucy An, David L Buckeridge, Antony Robert, James M G Tsui

**Affiliations:** Department of Experimental Medicine, McGill University, Montreal, QC H4A 3J1, Canada; Faculty of Medicine and Health Sciences, McGill University, Montreal, QC H3G 2M1, Canada; Department of Epidemiology and Biostatistics, McGill University, Montreal, QC H3A 1G1, Canada; Department of Emergency Medicine, McGill University Health Centre, Montreal, QC H4A 3J1, Canada; Department of Oncology, Division of Radiation Oncology, McGill University Health Centre, Montreal, QC H4A 3J1, Canada

**Keywords:** machine learning, race, bias, emergency department

## Abstract

**Objectives:**

Machine learning (ML) models are increasingly being developed to support healthcare delivery. However, concerns remain about their potential to perpetuate existing biases rooted in the data used to develop them. We aim to assess the impact of using race in predicting hospital admission probabilities for patients visiting the emergency department (ED).

**Materials and Methods:**

Data from the MIMIC-IV ED dataset were used to train2 ML models predicting hospital admission: one included race; the other did not. Differences in predicted admission probabilities were evaluated across racial groups under multiple validation conditions.

**Results:**

Including race as a model input was associated with meaningful differences in predicted admission probabilities for White (3.2%), Black (−1.5%), and Hispanic (−3.0%) patients, while minimal differences were observed for Asian (0.2%) and Other (0.5%) patients. These differences were associated with large Cohen’s *d* effect sizes in the baseline model for White (*d* = 1.00), Black (*d* = −1.23), and Hispanic (*d* = −1.35) patients. After balancing racial group prevalence, the effects persisted for White (1.22%; *d* = 1.22) and Hispanic (−1.00%; *d* = −1.00) patients.

**Discussion:**

These findings suggest that race is associated with differences in ML-based hospital admission predictions for ED patients, underscoring the need for caution when incorporating race into clinical prediction models and the importance of rigorous bias assessment.

**Conclusion:**

As race was associated with predictions, there is a crucial need to address underlying social factors and the use of broader, more equitable clinical data for ML model training.

## Background and significance

Machine learning (ML) has become an invaluable tool in medicine. Its application extends from disease prediction to imaging analysis and improves diagnostic accuracy, workflow efficiency, and healthcare delivery.^1–3^ As ML models are adopted into clinical care, they offer significant opportunities for improving patient outcomes but also present challenges. A key concern is that their effectiveness and equitable use depend on the quality and accuracy of their training data. If the underlying data are biased, these models may inadvertently reinforce existing healthcare biases and disparities.^4^^,^^5^ For example, historical treatment patterns may encode systemic biases, such that patients from marginalized groups receive different levels of care for similar clinical presentations, which can be learned and perpetuated by ML models.^6–8^ Documentation bias may also arise when clinical information is recorded inconsistently across patient groups during data acquisition, leading to differences in feature quality.^9^ Together, these biases in the data used to train ML may reflect historical patterns of care rather than underlying patient need and therefore cause models to systematically under- or overestimate risk for certain populations, thereby contributing to disparities in predicted outcomes.^4^^,^^10^^,^^11^

Healthcare disparities persist, disproportionately affecting marginalized racial and ethnic groups.^10^^,^^12^ While social determinants of health (SDoH), including access to care, socioeconomic status, and structural inequities, are major contributing factors, these factors are often incompletely captured in clinical datasets. As a result, ML models may implicitly learn their effects through proxy variables such as race, potentially contributing to disparities in predicted outcomes. Inequities are also driven by healthcare systems themselves.^13^ These include differences in access to care,^14^^,^^15^ and variation in treatment offered,^6^^,^^8^ leading to worse health outcomes for certain populations based on race, gender, disability, and other intersecting identities.^16^ As ML learns from real-world data shaped by SDoH and health inequities, models risk embedding systemic biases and thus, influencing predicted outcomes and treatment recommendations.

Algorithmic fairness aims to assess artificial intelligence (AI) models in order to identify and mitigate health disparities instead of propagating them.^17^ Concern is raised on how AI models can affect underrepresented communities as biases embedded in data acquisition can permeate through ML model, leading to inequitable outcomes in clinical settings.^17–19^ Although awareness of algorithmic fairness is growing, much of the recent work focuses on improving the technical performance of ML models, while neglecting the sociotechnical dimension.^20^ Indeed, ML can be leveraged as a tool to investigate how socioeconomic factors influence healthcare outcomes and provide a better understanding of biases embedded in clinical decision-making.

The MIMIC-IV dataset, a publicly available dataset containing extensive clinical data from the emergency department (ED), provides an opportunity to evaluate ML model performance across diverse populations. Previous studies done using this dataset have shown that while models trained on broad racial categories may appear fair, they often fail to capture meaningful subgroup disparities.^21^ These biases can be amplified when models rely heavily on demographic features such as race and sex, which can contribute to unfair clinical predictions and treatment modalities.^22^ Work on the MIMIC-IV dataset has explored the relationship between race and clinical outcomes, including disparities between White and racialized groups.^23^ However, to our knowledge, much of the existing literature has focused on overall model performance or classification outcomes, with less attention to how the inclusion of race as an input feature may systematically shift predicted probabilities across racial groups. In particular, differences in the magnitude and direction of these shifts, as well as their corresponding effect sizes, remain underexplored.

## Objective

Our research aimed to assess the impact of using race as an explicit variable in predicting hospital admission probabilities for patients visiting the ED, using the MIMIC-IV dataset. We first developed a model with performance comparable to benchmarks reported in the literature, and then evaluated how the inclusion of race affected predicted probabilities across racial groups, with a focus on the magnitude and direction of these differences.

## Materials and methods

### Formulation of hypothesis

Machine learning models can be trained to take the same types of input information available to clinicians in the ED and produce a probability of admission. This probability of admission can be expressed as f(Z)


(1) f(Z)=P(Y=1 | Z) + ∈

where *Z* is the full set of input variables, *Y*∈ {0,1} denotes the binary admission decision, and ε is an irreducible noise term accounting for inherent uncertainty in the decision-making process. However, since we do not have access to the full set of variables *Z*, we instead work with a subset of observed variables which constitutes the input to our model, denoted as *X*⊆*Z*. Additionally, we consider race, represented as *R*, as a variable of interest. Our goal is to evaluate whether the inclusion of race as a model input is associated with differences in predicted admission probabilities by comparing models trained with and without *R*.

To this end, we define 2 multilayer perceptron (MLP) models trained on the same dataset but with different input feature sets:


(2) $$f_{1}(X)=\hat{P_{1}}=P\left(Y=1|X\right)\;+\;\in_{1},\;\mathrm{Model}\;1\;\mathrm{without}\;\mathrm{race}$$

(3) $$f_{2}(X,R)=\hat{P_{2}}=P\left(Y=1|X,R\right)\;+\;\in_{2},\;\mathrm{Model}\;2\;\mathrm{including}\;\mathrm{race}$$

where *X* represents all observed input features excluding race, *R*. $\hat{P_{1}}$ and $\hat{P_{2}}$ are the predicted probabilities of admission for the model trained with without race and with race, respectively.

Given these 2 models, we aim to explore 2 key questions, whether the inclusion of race lead to a meaningful difference in predicted admission probability and if so, the magnitude of variation across racial groups. It is important to note that with a large sample size (>350 000 data points), even minimal differences will reach statistical significance. Therefore, to assess the practical impact of race on predictions, we calculated the *Cohen’s d* effect size (see eqn (6)). Finally, while this is a classification task, we adopted clinical parlance and referred to it as prediction.

**Figure 1.**
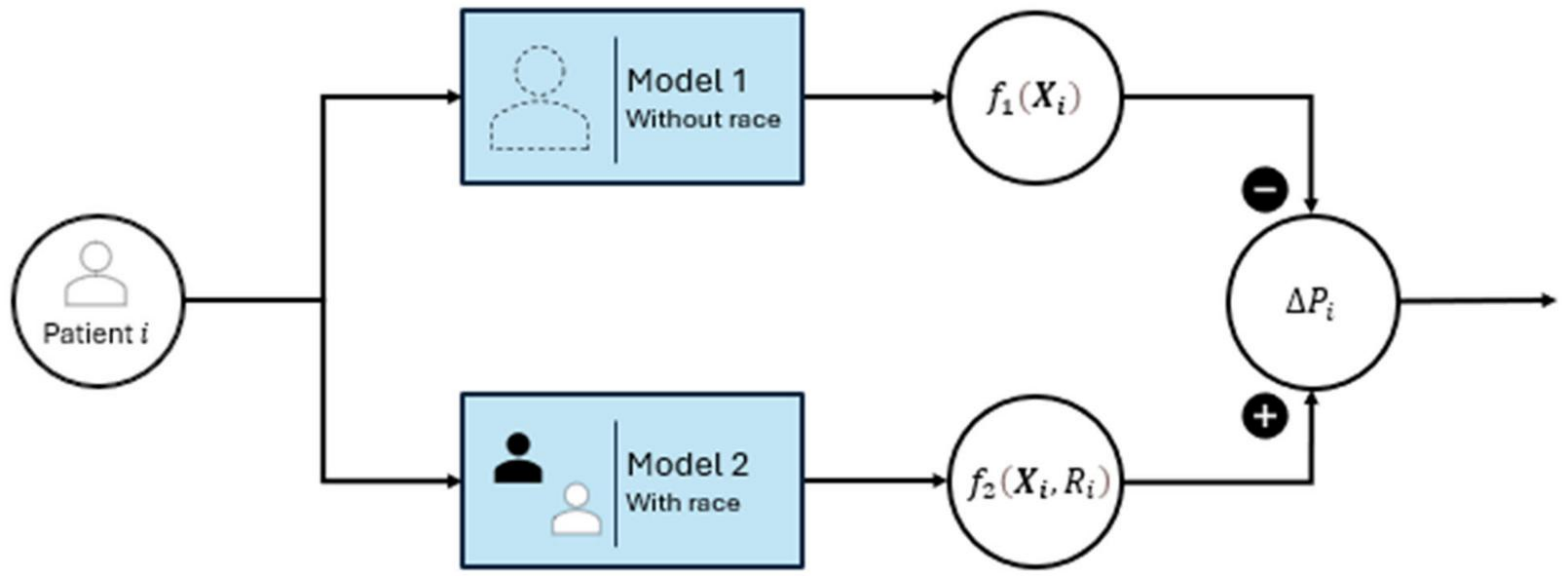
Framework to assess the impact of race on model prediction—The probability of admission predicted for patient i using the model trained without race, $f_{1}(X_{i})$, is subtracted from the probability predicted by the model trained with race, $f_{2}(X_{i},R_{i})$, to assess the impact of race on the model’s output, $\Delta P_{i}$.

We defined the probability of admission using input variables excluding race as $f_{1}(X_{i})$, and including race as $f_{2}(X_{i},R_{i})$, then defined the change in predicted probability for each individual i before and after race is included (Figure 1):


(4) $$\Delta P_{i}=f_{2}(X_{i},R_{i})-f_{1}(X_{i})$$

If $\Delta P_{i}$ approaches zero for most individuals, then race does not meaningfully affect predictions. If $\Delta P_{i}$ is systematically positive or negative for certain racial groups, then it would suggest that race plays a role in the model’s admission decision, with a positive value indicating a positive association between the individual’s race and the likelihood of admission.

To further assess how race is associated with model predictions, we compared the differences in the predicted admission probabilities $\Delta P_{i}$, and averaged these differences across individuals within each racial group:


(5) $$\frac{\sum_{i}^{k}\Delta P_{i}^{k}}{n^{k}}$$

Here, i represents individual patients, k denotes the racial group to which patient i belongs, and $n^{k}$ is the number of patients in racial group k. We then repeated the training and inference on the validation set 50 times to minimize noise in weight initialization and training, along with equalizing weights before training and using the same batches in backpropagation.

To obtain the effect size, we employed *Cohen’s d* test:


(6) d= ∑if2(Xi,Ri) n-∑i f1(Xi) ns

where n is the number of patients, s is the pooled standard deviation, and a value of d <0.2, around 0.5, and >0.8 represents small, medium, and large effects, respectively.

### Dataset

This study was performed using the MIMIC-IV dataset, which consists of 425 087 ED visits from 2011 to 2019 from patients at the Beth Israel Deaconess Medical Center in Boston, MA, United States.^24^

### Preprocessing

Time of day of arrival was normalized to a value between 0 and 1. Patient age was included as continuous variable. Categorical variables were one-hot encoded. Sex included male and female, and mode of arrival was grouped into ambulance and walk-in (other modes such as helicopters and unknown were negligible, thus discarded). Race was originally documented as free text, often with extended descriptors (eg, “HISPANIC/LATINO—BUCAN” or “BLACK/CAP VERDEA”). For consistency, entries were filtered to the first word of the description and subsequently grouped into 5 categories: White, Black, Hispanic, Asian, and Other.

Clinical numerical variables included: temperature, heart rate, respiration rate, oxygen saturation, systolic blood pressure, diastolic blood pressure, and triage acuity. A predefined dictionary was used to establish physiologically feasible ranges removing extreme outliers: *feasible_ranges* = {*'temperature’*: [96, 104], *'heart rate’*: [20, 300], *'respiratory rate’*: [5, 80], *'o2 saturation’*: [70, 100], *'systolic blood pressure’*: [50, 250], *'diastolic blood pressure’*: [30, 150], *'triage acuity’*:[1, 5]}.

The patient’s primary complaint at triage was obtained from the text in the “*chiefcomplaint*” column. The text was preprocessed by converting all characters to lowercase, followed by Term Frequency-Inverse Document Frequency (TF-IDF) vectorization. A minimum word frequency threshold of 10 was applied to filter out infrequent terms, and the final feature size was set to 1018. To prevent data leakage, the vectorizer was fit on the training set and subsequently used to transform the validation set. All rows containing missing values or vital signs values that fell outside of physiologically feasible ranges were excluded.

The dataset was split into training and validation sets using a 70:30 ratio, and the same split was used for all reruns to ensure consistency.

### Outcome

The primary outcome variable of admission status was directly extracted from the disposition column of the dataset. To create a binary classification, patient outcomes were characterized as admitted or nonadmitted/discharged.

### Model

**Figure 2.**
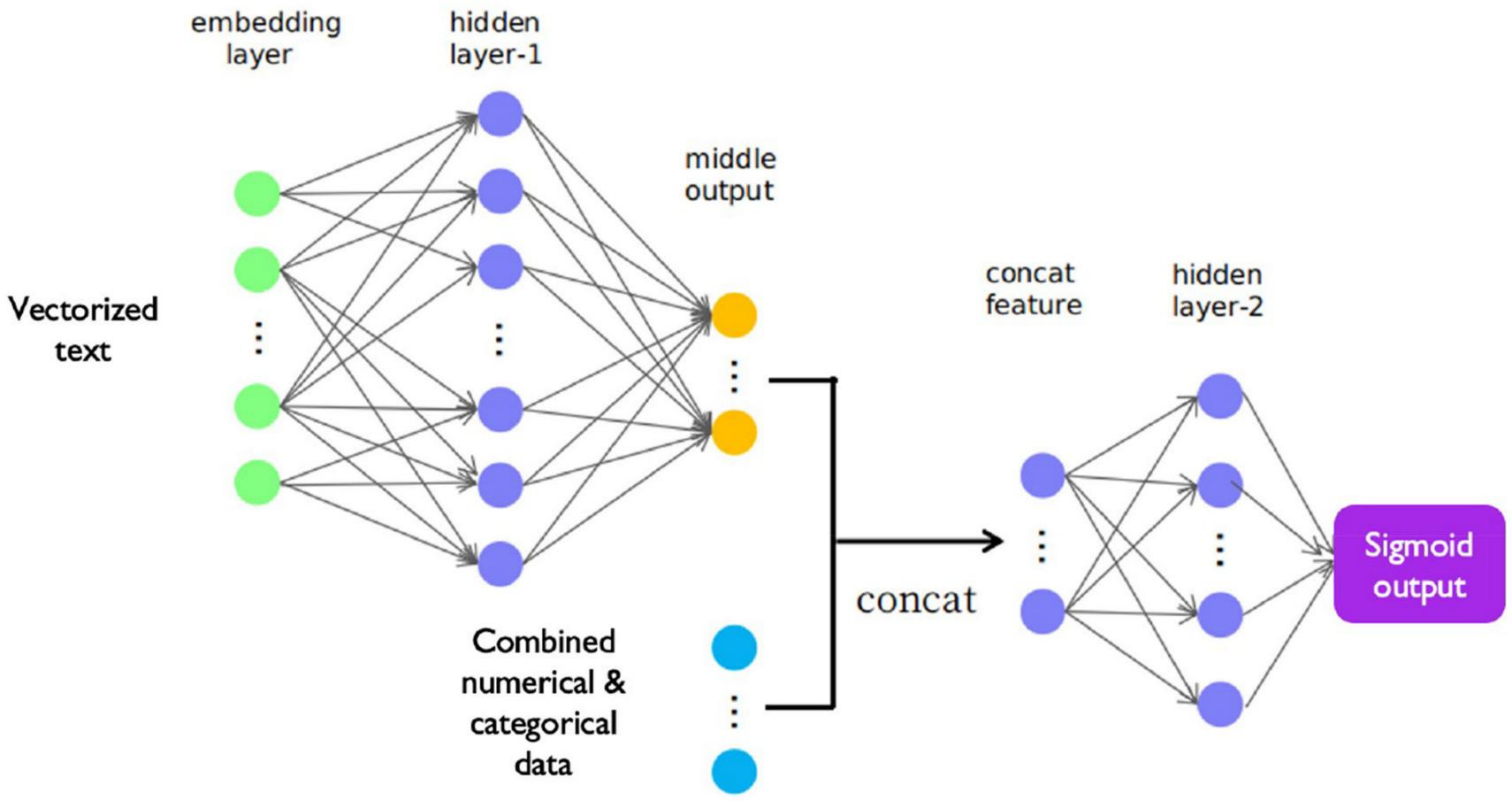
Multilayer perceptron model architecture. Free-text data from the chief complaint are first vectorized using TF-IDF and passed through a neural network to generate text embeddings. These embeddings are then concatenated with numerical and categorical data and fed into a classification MLP to predict hospital admission. Abbreviation: TF-IDF, Term Frequency-Inverse Document Frequency.

The classification model was implemented using an MLP architecture (Figure 2). Frequency encoded text features from the chief complaint were fed into the MLP input layer, followed by 2 hidden layers. These layers learned a condensed representation of the textual feature space. This text-based representation was then concatenated with the remaining clinically variables, comprising both numerical and one-hot encoded categorical features (13 when race was excluded, 18 when included; Table S1). This combined feature set was then passed through an additional hidden layer, followed by an output layer with a sigmoid function for classification to produce a binary prediction for hospital admission (see “Hyperparameters” section).

The model was trained using binary cross-entropy loss. Two models were trained using the same training and validation dataset: one model included race as input variable and the other did not. Model performance included the sensitivity, specificity, area under the curve (AUC) of the receiver operating characteristics (ROC), and accuracy. Performance metrics and analyses were reported solely on the validation dataset.

Model evaluation also included calibration curves and interpretability analysis using SHapley Additive exPlanations (SHAP) values. For features represented by one-hot encoding, SHAP contributions were summed across categories, and for text-based inputs, contributions from the embedded feature vectors were summed to obtain the overall contribution of text.

We chose not to balance the dataset by admission outcome, as doing so could introduce bias related to race into the model. However, in additional analyses, we addressed imbalance in racial group representation by constructing a balanced dataset through random upsampling of non-White groups so that each group had the same number of individuals as the White group. This upsampling was performed separately for the training and validation sets. The model was then retrained on the balanced dataset and evaluated on the corresponding validation set.

To further assess whether the observed effects were attributable to the race variable itself, we conducted an additional analysis in which race labels were randomly permuted at inference time. Model predictions were then evaluated while preserving the true race labels for reporting.

Descriptive statistics were used to report the distribution of racial groups and admission outcomes.

### Hyperparameters

Five primary hyperparameters were tuned: batch size, number of hidden layers, hidden unit sizes, number of training epochs, and dropout rate. The Adam optimizer was used with a fixed learning rate of 0.001 to ensure stable convergence. The selected hyperparameters included a batch size of 32, a first hidden layer with 64 units, a second hidden layer with 32 units for text embedding, a final layer with 32 units following the concatenation step. Random search was used for hyperparameter tuning, and the selected configuration was chosen for its balance of predictive accuracy and computational practicality.

### Controlling for training variability

To ensure that observed differences in predictions were truly attributable to the inclusion of race as a variable, several measures were taken to control for noise. Since model training can be sensitive to initialization, even identically structured models can converge to different solutions. This variability could create the false impression of a discrepancy in predictions, potentially leading to incorrect conclusions about the impact of race on the model’s decision. To account for this, we implemented 2 controls. First, the weights were controlled for both models. The model that excluded race as input was initialized using Glorot initialization.^25^ To ensure consistency, the model that included race was then initialized with the same weights to match with the model without race, except for the additional parameters corresponding to the race input, which were randomly initialized. Next, to control for the direction of optimization, both models were trained on the exact same set of batches. This ensured that if the models were truly equivalent, they would receive identical gradients on every batch, minimizing variability due to stochastic differences in training.

Finally, there was also inherent noise in the training process, as the same model trained on the same dataset with different initializations and batch orders may converge to different local optima. To account for this variability, we conducted 50 reruns using identical training and validation sets. This approach allowed us to quantify the variability across runs. By averaging the predictions across all runs, we obtained a more robust estimate of the model’s expected output on the validation set, reducing the influence of stochastic noise.

## Results

The total of 425 087 ED visits from the MIMIC-IV dataset was reduced to 376 211 after preprocessing and exclusion of all rows containing missing values or vital signs values outside of the physiological range. Race and ethnicity in MIMIC-IV are recorded as a single combined variable, with 33 unique entries identified (Table S2). During preprocessing, race was grouped using the first word of the description, resulting in 5 major categories: White, Black, Hispanic, Asian, and Other. Categories beyond these groups were negligible in size and were therefore combined into the “Other” category. The full list of unique race entries in the MIMIC-IV dataset, as well as the distribution of race after grouping by the first word of the descriptor, are shown in Table S2 and Figure S1, respectively. The final distribution of race was 4.5%, 6.3%, 8.3%, 22.4%, and 58.5% for Asian, Other, Hispanic, Black, and White, respectively. The primary outcome was admission status, and our dataset had a proportion of 37.2% of ED visit ending up with an admission. Training and validation sets were randomly split (Table 1).

**Table 1.** Distribution of racial groups and admission outcomes by racial group in the full cohort, training set, and validation set.

Racial group	All (*n* = 376 211)	Training (*n* = 263 347)	Validation (*n* = 112 864)
White	220 164 (58.5%)	153 964 (58.5%)	66 200 (58.7%)
Admitted	95 631 (43.4%)	66 833 (43.4%)	28 798 (43.5%)
Not admitted	124 533 (56.6%)	87 131 (56.6%)	37 402 (56.5%)
Black	84 087 (22.4%)	59 014 (22.4%)	25 073 (22.2%)
Admitted	24 059 (28.6%)	16 946 (28.7%)	7113 (28.4%)
Not admitted	60 028 (71.4%)	42 068 (71.3%)	17 960 (71.6%)
Hispanic	31 392 (8.3%)	21 973 (8.3%)	9419 (8.3%)
Admitted	7847 (25.0%)	5487 (25.0%)	2360 (25.1%)
Not admitted	23 545 (75.0%)	16 486 (75.0%)	7059 (74.9%)
Other	23 700 (6.3%)	16 493 (6.3%)	7207 (6.4%)
Admitted	7210 (30.4%)	5030 (30.5%)	2180 (30.2%)
Not admitted	16 490 (69.6%)	11 463 (69.5%)	5027 (69.8%)
Asian	16 868 (4.5%)	11 903 (4.5%)	4965 (4.4%)
Admitted	5253 (31.1%)	3711 (31.2%)	1542 (31.1%)
Not admitted	11 615 (68.9%)	8192 (68.8%)	3423 (68.9%)

We trained a model to predict admission without race as an input feature. The validation set achieved a sensitivity of 0.613, specificity of 0.859, AUC of 0.838, and accuracy of 0.768. We also trained the model to predict admission outcome for each visit, including race as an input variable. The validation set achieved a sensitivity of 0.592, specificity of 0.871, AUC of 0.839, and accuracy of 0.768.

We then evaluated changes in predicted admission probabilities across racial groups by comparing models with and without race as an input feature (Figure 1). The mean percent change in predicted admission probabilities for each racial group was 1.9%, −3.1%, −5.0%, −1.0%, and −1.2% for White, Black, Hispanic, Other, and Asian individuals, respectively (Table 2), with corresponding effect sizes of 1.00, −1.23, −1.35, −0.63, and −0.67 (Table S3). The effect sizes were considered large for White, Black, and Hispanic individuals, and moderate for Other and Asian groups.

**Table 2.** Mean percent difference in predicted admission probabilities across racial groups in the baseline, balanced, and randomized-balanced validation datasets.

	Mean percentage difference		
	Baseline (%)	Balanced (%)	Randomized balanced (%)
White	1.9	3.2	0.2
Black	−3.1	−1.5	0.3
Hispanic	−5.0	−3.0	0.0
Other	−1.0	0.2	0.8
Asian	−1.2	0.5	−0.3

After balancing the racial groups, resulting in 153 964 and 66 200 individuals for each racial group in the training and validation set, respectively, the mean percent changes were 3.2%, −1.5%, −3.0%, 0.2%, and 0.5%, for White, Black, Hispanic, Other, and Asian individuals, respectively, with corresponding effect sizes of 1.22, −0.65, −1.00, 0.14, and 0.23. The trend persisted for White, Black, and Hispanic groups, while effect sizes for Other and Asian groups became negligible.

Finally, using the balanced dataset, we randomized race labels across patients prior to model input, while still reporting outcomes by original race. This yielded mean percent changes of 0.2%, 0.3%, 0.0%, 0.8%, and −0.3%, for White, Black, Hispanic, Other, and Asian individuals respectively, with corresponding negligible effect sizes of 0.13, 0.22, −0.03, 0.52, and −0.18.

**Figure 3.**
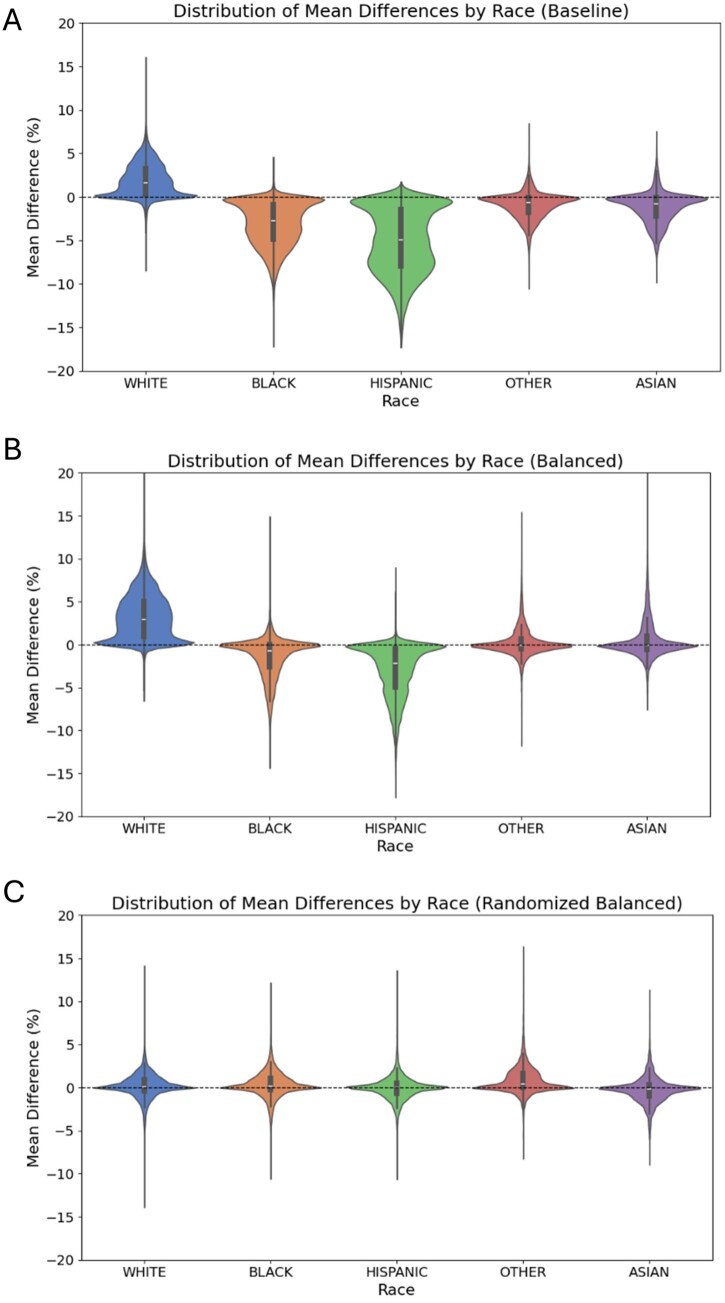
Distribution of mean percentage change in predicted admission probabilities across racial groups. (A) Baseline—consists of the original dataset after preprocessing. (B) Balance—all racial groups were randomly up sampled to ensure equal distribution. (C) Randomized balanced—race labels were randomly reassigned at inference, but results were reported according to each patient’s original race.

To further characterize these differences beyond mean values, we examined the full distribution of Δ*P* (difference in predicted admission probability between models with and without race) across racial groups (Figure 3). For White patients, the distribution is centered near zero but exhibits a pronounced upward tail, indicating that while most predictions remain similar, a substantial subset of cases shows higher predicted admission probabilities when race is included (Figure 3A). In contrast, the distributions for Black and Hispanic patients show the opposite pattern compared to White patients, with shifts toward lower values, indicating lower predicted probabilities when race is included. These patterns persist in the class-balanced analysis (Figure 3B) but are no longer observed when race labels are randomized (Figure 3C), where the distributions are centered around zero and resemble a Gaussian (normal) distribution across groups.

To better contextualize these differences in predicted probabilities in terms of model performance, we further examined sensitivity and specificity across racial groups (Table 3). When stratified by race, model sensitivity was highest among White patients, followed by Asian and Other groups, and was substantially lower for Black and Hispanic patients. The most notable changes associated with the inclusion of race as a model input were decreases in sensitivity for Black patients (0.535-0.432) and Hispanic patients (0.489-0.389), representing a 10.3% and 9.9% reduction, respectively, while changes for other racial groups were comparatively smaller (−3.6% to 0.7%).

**Table 3.** Performance metrics by racial group.

Group	Model	Accuracy	Sensitivity	Specificity	AUROC
Full dataset	No race	0.7675	0.6134	0.8589	0.8381
	With race	0.7675	0.5924	0.8713	0.8388
White	No race	0.7497	0.6469	0.8289	—
	With race	0.7483	0.6542	0.8207	—
Black	No race	0.7861	0.5352	0.8855	—
	With race	0.7877	0.4319	0.9286	—
Hispanic	No race	0.7959	0.4886	0.8987	—
	With race	0.8039	0.3894	0.9425	—
Other	No race	0.8069	0.5839	0.9035	—
	With race	0.8059	0.5523	0.9159	—
Asian	No race	0.8002	0.5804	0.8992	—
	With race	0.7974	0.5447	0.9112	—

Abbreviation: AUROC, area under the receiver operative characteristics.

**Figure 4.**
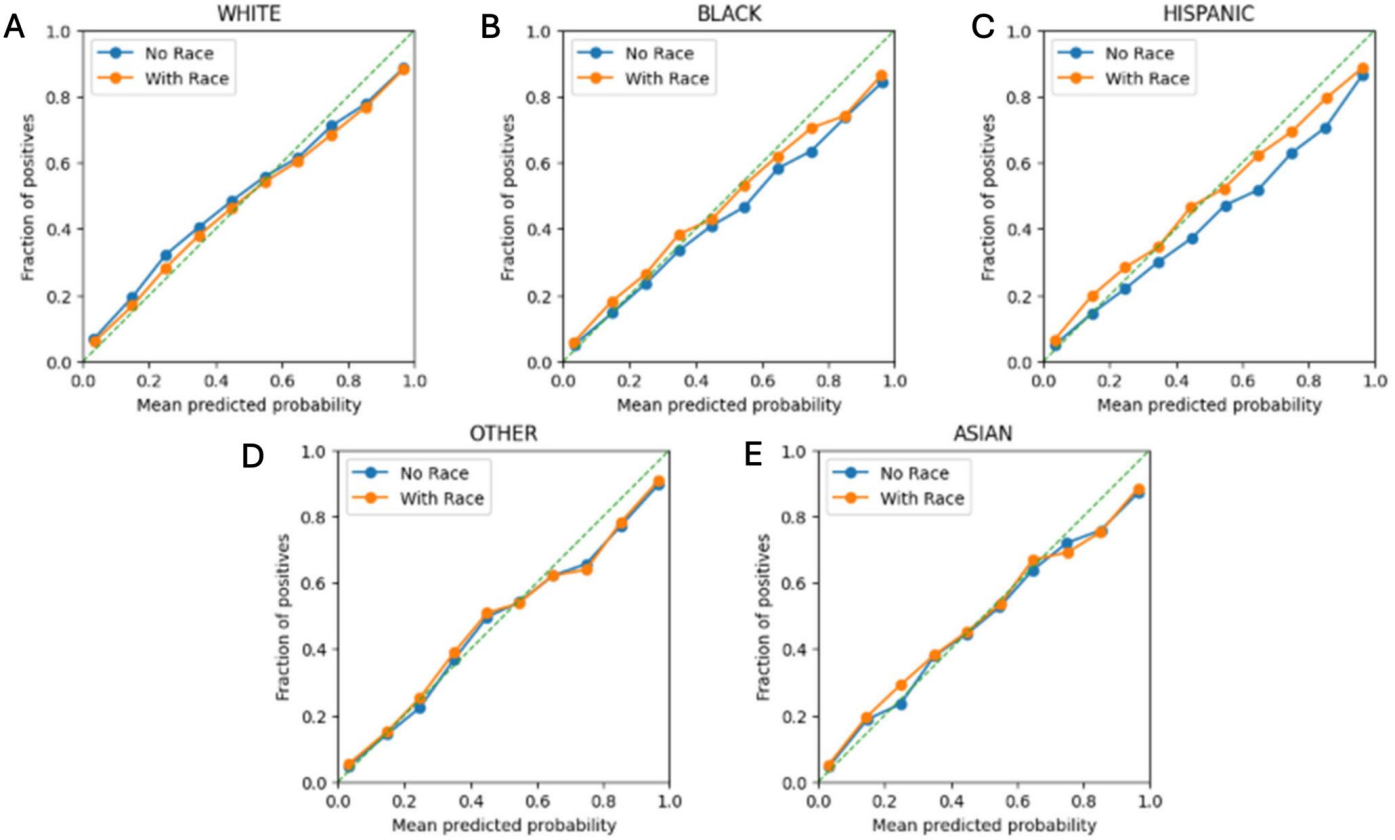
Calibration curves for the model trained without race (blue) and with race (orange) across racial groups—(A) White, (B) Black, (C) Hispanic, (D) Other, and (E) Asian.

The calibration curves for the models trained with and without race across racial groups demonstrated generally good calibration (Figure 4) with the calibration lines following the diagonal reference lines across all groups. Curves tended to dip below the diagonal at higher predicted probabilities, suggesting that the model systematically overestimated risk in patients with the highest predicted probabilities. For the Hispanic group (and the Black subgroups to a lesser extent), the curves for the model trained with race, except for the extreme ends of the probability spectrum (near 0 or 1), consistently shifted leftward compared to the model without race, suggesting the inclusion of race was associated with lower predicted risks at intermediate probability levels. For the White group, the effect was reversed: the inclusion of race shifted the calibration curve slightly to the right, suggesting higher predicted risks at intermediate probability levels. Although this shift was small, it was notable that, apart from the extremes, all points moved rightward with the inclusion of race. In contrast, for the Asian and Other groups, the calibration curves intersected each other, indicating no consistent directional shift with or without race.

**Figure 5.**
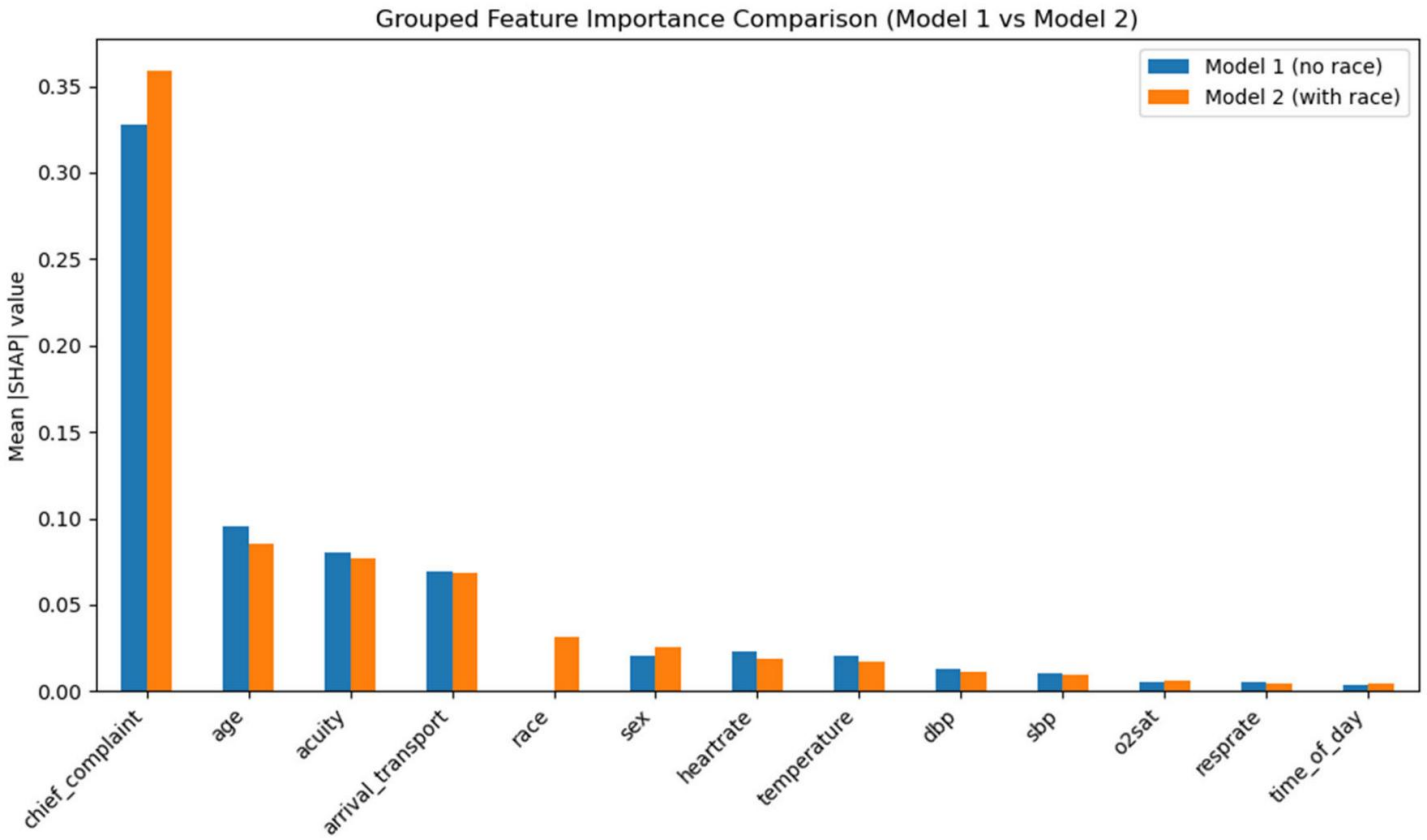
SHAP-based grouped feature importance comparison for models trained with and without race.

SHapley Additive exPlanations analysis was conducted to evaluate feature contributions for the models trained with and without race (Figure 5). In both models, chief complaint was by far the most influential predictor, followed by age, acuity, and mode of arrival, with relatively smaller contributions from physiologic variables such as heart rate, blood pressure, and oxygen saturation. The addition of race was associated with only a modest contribution to the model. However, the inclusion of race slightly increased the importance of several existing top features. Overall, the feature importance distribution was consistent across the 2 models, indicating that predictive performance was primarily driven by clinical presentation and demographics rather than by race.

## Discussion

Our model trained with race achieved an AUC of 0.842, which is comparable to similar previous work with AUC values of 0.846^26^ and 0.826^27^ for predicting admission using the same MIMIC-IV ED dataset. Furthermore, our findings show that race was associated with differences in the predicted probability of admission from the ED for Hispanic, Black, and White patients, while there are no meaningful effects for Asian and Other races patients. Even after controlling for class imbalance, the effect sizes of −1.0 for Hispanic, −0.65 for Black, and 1.22 for White suggest that racial disparities persist despite adjustment. Although race had modest importance (Figure 5), even small effects can systematically shift predicted probabilities across large populations, leading to meaningful differences.

These findings have important clinical safety implications. These differences in predicted probabilities translate into meaningful differences in model performance across subgroups. The observed reductions in sensitivity for Black and Hispanic patients when race was included as an input feature suggest that models incorporating race may be more likely to underestimate admission needs in these populations. In a clinical setting, this could translate into a higher risk of missed or delayed admissions, potentially exacerbating existing disparities in care.

These findings align with prior literature showing how a variety of factors influence the experiences of marginalized patients within the healthcare system. For example, Hispanic patients may delay seeking care for fear of discrimination or language barriers,^28^^,^^29^ while Black patients’ experiences are often influenced by medical racism and mistrust in the medical system.^30^ Clinical decision-making itself is not immune to bias^27^; studies show that pain is often identified earlier in White patients compared to Black patients,^31^ and ethnic minority children are more often misdiagnosed.^32^ Such patterns suggest that race, while not a physiological variable, reflects broader structural and SDoH that influence both patient behavior and provider judgment. These patterns may be reflected and potentially perpetuated in ML models trained on such historical clinical data.

While race may be associated with differences in the model’s admission prediction probability, it is unlikely to be the sole driver. Differences in insurance coverage, income, education, housing, and the ability to take time off work, all of which rarely considered in clinical algorithms, likely contributed significantly to patient admission decision.^33–35^ At the same time, implicit biases among healthcare professionals are well documented^6^^,^^9^ and may influence clinical decision, reinforcing disparities.^36^ Because algorithms learn from such human decisions, they risk perpetuating or even amplifying these same biases.^37^

Indeed, racial bias has been well documented across various ML applications in health care, with current fairness metrics proven insufficient to capture the full picture.^10^ As such, widely used algorithms have underestimated care needs for Black patients,^11^ and natural language processing studies reveal that Black patients’ records are more likely to contain negative descriptors.^9^ From inequitable resource allocation to performance disparities across subgroups, studies reveal that biases can be embedded at multiple stages of predictive clinical models.^11^^,^^38^^,^^39^

Recent work has explored marginal debiasing, an approach that adjusts model performance across individual protected attributes to promote fairness.^40^ It showed that fairness metrics, such as calibration error, improve when race and intersectional subgroups are considered. However, they did not examine how these disparities translate into differences in admission outcomes. Similarly, studies using other databases developed ML models to predict hospital admissions from the ED with race included as part of a broader demographic set, but not examined independently for its specific contribution to model performance.^41^^,^^42^

Some healthcare AI guidelines have begun to acknowledge the importance of addressing racial and ethnic bias. MINIMAR^43^ explicitly states that patient demographic characteristics, including race and ethnicity, should be 1 of the 4 essential components of AI reporting standards. PROBAST + AI^44^ addresses the risk of bias in model evaluation and includes criteria to ensure that test datasets are representative of the training population, including with respect to race and ethnicity. While both guidelines emphasize the need for demographic representativeness in datasets, they do not explicitly address how these variables may influence model decision-making or performance and fail to explicitly highlights the importance of including SDoH as input variables.

Without thoroughly evaluating these tools, there is a risk of under- or overestimating admission need, as models might overlook important socioeconomic factors that clinicians typically consider in their patient assessment. In clinical practice, caution is warranted when implementing ML models as there could be biases incorporated unknowingly and only become apparent when specific factors, such as race as explored in this study, are isolated and looked at.

This study aligns with previous research demonstrating the presence of racial bias in clinical documentation and decision-making.^6^^,^^8^ Our work complements previous studies looking at existing biases by quantitatively demonstrating how these biases affect admission probability. While our findings illustrate that racial disparities are embedded within historical clinical data and can be learned by ML models, we recommend that race not be included in pathophysiology-based clinical prediction models without first evaluating its impact and the risk of propagating unintended biases.

There are several limitations to our study. The study relies on the MIMIC-IV dataset, which is derived from Beth Israel Deaconess Medical Center, a large academic tertiary care center in Boston, MA, United States, that serves a diverse urban population with substantial racial, ethnic, and socioeconomic heterogeneity. However, as the dataset is limited to a single hospital center, the generalizability of these findings to other geographic and clinical contexts may be limited. The dataset also does not capture the full range of socioeconomic factors that may influence decision-making, and insurance coverage information is incomplete. It is also unknown whether the admission decision came from the healthcare provider or the patient, as it would be possible for patients to insist on admission or refuse admission despite healthcare professional recommendation.

Additionally, race was derived from a variable in the MIMIC-IV dataset that combines race and ethnicity, without detailed documentation on how these data were originally collected beyond routine clinical care and was further aggregated during the deidentification process. In our preprocessing, we extracted the primary race descriptor (ie, the first word of the race field) to categorize patients into 5 groups. This approach may oversimplify the distinction between race and ethnicity and conflate the 2 constructs, particularly given the unique status of Hispanic as an ethnicity rather than a race, and may failed to capture multiracial or overlapping identities, thereby introducing potential misclassification. Furthermore, the “Other” category is highly heterogeneous and difficult to interpret. Despite the overall large sample, substantial variability within this group (Figure S1), precluded further stratification into more specific categories.

From a modeling perspective, the study focused on predicting admission probability at triage for simplicity of analysis, which may overlook incomplete medical histories or transient symptoms, potentially reducing the prediction accuracy. Additionally, as the goal of this study was not to optimize generalizability but to uncover potential biases in admission prediction using a ML model and a separate holdout test set was not deemed necessary.

In this context, we selected a relatively simple MLP architecture to enable a controlled comparison, as it allows integration of high-dimensional text features with structured variables in a single end-to-end framework and can capture nonlinear relationships. While simpler models such as logistic regression assume linear relationships, methods such as gradient boosting or support vector machines can capture nonlinear patterns but often require additional hyperparameter tuning and feature engineering. In contrast, the MLP provides a flexible yet controlled end-to-end approach for integrating high-dimensional inputs. Importantly, using a consistent model architecture and training procedure across experiments allows observed differences in predictions to be attributed specifically to the inclusion of race.

During model development, individual encounters were treated as independent observations. As a result, some patients may have contributed multiple encounters across training and validation sets, introducing potential patient-level dependence and overlap between splits. This overlap was associated with higher observed admission rates in the validation cohort among encounters from patients seen during training (Figure S2), likely reflecting underlying differences in patient risk profiles. However, this pattern was consistently observed across all racial groups, suggesting that it represents a broad effect rather than one specific to any single group. We also examined the distribution of racial groups before and after exclusion of entries with missing or out-of-range values and found that it remained largely unchanged, suggesting no major differential exclusion across racial groups at the aggregate level (Figure S3). However, we acknowledge that we cannot determine whether the missing data were missing at random.

Lastly, our dataset was not well balanced in terms of admission rates across racial groups. The proportion of patients admitted was significantly higher for White patients compared to other racial groups, with the largest difference observed between White and Hispanic of 18.4% in the training set. As a result, the model may have learned to prioritize specificity over sensitivity for Hispanic patients. Furthermore, despite the lower admission rate for Hispanic patients, the model still achieved higher overall accuracy than for White patients, and this finding persisted even when race was not used as an input feature.

## Conclusion

The difference in our models trained with or without race shows that race was associated with variation in admission predictions. These associations should not be interpreted as causal, as race likely serves as a proxy for unmeasured SDoH and structural factors influencing both patient presentation and clinical decision-making. Building on these findings, future efforts should be made to explore the underlying socioeconomic factors that may be associated with race, developing more equitable clinical tools, and collect more comprehensive data to reflect real-life clinical decision as opposed to a physiological snapshot. Indeed, ML models should not only be trained on physiological data when, in an ED setting, many different factors come into play to influence the outcome decision.

In the absence of complete data on the SDoH, our findings suggest that including race as a predictive variable may introduce unintended biases, including potential underestimation of admission probability for certain groups. This raises important ethical and methodological considerations. Race may therefore be more appropriately used for fairness auditing rather than direct prediction; however, its use remains part of an ongoing debate in clinical AI fairness research.

## Supplementary Material

ooag157_Supplementary_Data

## Data Availability

The datasets generated and/or analyzed during the current study are available in the MIMIC-IV dataset through credentialed access: https://physionet.org/content/mimiciv/2.2/. Experiments’ data can be reproduced through placing the data under a mimic-iv-2.2/folder in the provided code repository. The underlying code for this study is available here: https://github.com/alicoppe/racial-bias-in-ed-prediction.

## References

[1] Akinrinmade AO , Adebile TM, Ezuma-Ebong C, et al Artificial intelligence in healthcare: perception and reality. Cureus. 2023;15:e45594. 10.7759/cureus.45594 37868407 PMC10587915

[2] Currie G , Hawk KE, Rohren E, Vial A, Klein R. Machine learning and deep learning in medical imaging: intelligent imaging. J Med Imaging Radiat Sci. 2019;50:477-487. 10.1016/j.jmir.2019.09.005 31601480

[3] Gala D , Behl H, Shah M, Makaryus AN. The role of artificial intelligence in improving patient outcomes and future of healthcare delivery in cardiology: a narrative review of the literature. Healthcare. 2024;12:481. 10.3390/healthcare12040481 38391856 PMC10887513

[4] Barton M , Hamza M, Guevel B. Racial equity in healthcare machine learning: illustrating bias in models with minimal bias mitigation. Cureus. 2023;15:e35037. 10.7759/cureus.35037 36942183 PMC10023594

[5] Fu J , Reid SA, French B, et al; COVID-19 and Cancer Consortium (CCC19). Racial disparities in COVID-19 outcomes among Black and White patients with cancer. JAMA Netw. Open. 2022;5:e224304. 10.1001/jamanetworkopen.2022.4304 35344045 PMC8961318

[6] FitzGerald C , Hurst S. Implicit bias in healthcare professionals: a systematic review. BMC Med Ethics. 2017;18:19. 10.1186/s12910-017-0179-8 28249596 PMC5333436

[7] Hall WJ , Chapman MV, Lee KM, et al Implicit racial/ethnic bias among health care professionals and its influence on health care outcomes: a systematic review. Am J Public Health. 2015;105:e60-e76. 10.2105/AJPH.2015.302903 PMC4638275 26469668

[8] Macias-Konstantopoulos WL , Collins KA, Diaz R, et al Race, healthcare, and health disparities: a critical review and recommendations for advancing health equity. West J Emerg Med. 2023;24:906-918. 10.5811/WESTJEM.58408 37788031 PMC10527840

[9] Cobert J , Mills H, Lee A, et al Measuring implicit bias in ICU notes using Word-embedding neural network models. Chest. 2024;165:1481-1490. 10.1016/j.chest.2023.12.031 38199323 PMC11317817

[10] Huang J , Galal G, Etemadi M, Vaidyanathan M. Evaluation and mitigation of racial bias in clinical machine learning models: coping review. JMIR Med Inform. 2022;10:e36388. 10.2196/36388 35639450 PMC9198828

[11] Obermeyer Z , Powers B, Vogeli C, Mullainathan S. Dissecting racial bias in an algorithm used to manage the health of populations. Science. 2019;366:447-453. 10.1126/science.aax2342 31649194

[12] Dickman SL , Gaffney A, McGregor A, et al Trends in health care use among Black and White persons in the US, 1963-2019. JAMA Netw Open. 2022;5:e2217383. 10.1001/jamanetworkopen.2022.17383 35699954 PMC9198752

[13] Webster CS , Taylor S, Thomas C, Weller JM. Social bias, discrimination and inequity in healthcare: mechanisms, implications and recommendations. BJA Educ. 2022;22:131-137. 10.1016/j.bjae.2021.11.011 35531078 PMC9073302

[14] Beroukhim G , Mahabamunuge J, Pal L. Racial disparities in access to reproductive health and fertility care in the United States. Curr Opin Obstet Gynecol. 2022;34:138-146. 10.1097/GCO.0000000000000780 35645012

[15] McClintock HF , Kurichi JE, Barg FK, et al Health care access and quality for persons with disability: patient and provider recommendations. Disabil Health J. 2018;11:382-389. 10.1016/j.dhjo.2017.12.010 29325927

[16] National Academies of Sciences, Engineering, and Medicine, Health and Medicine Division, Board on Population Health and Public Health Practice, and Committee on Community-Based Solutions to Promote Health Equity in the United States. Communities in Action: Pathways to Health Equity. National Academies Press (US); 2017. 28418632

[17] Chen RJ , Wang JJ, Williamson DFK, et al Algorithmic fairness in artificial intelligence for medicine and healthcare. Nat Biomed Eng. 2023;7:719-742. 10.1038/s41551-023-01056-8 37380750 PMC10632090

[18] Agarwal R , Bjarnadottir M, Rhue L, et al Addressing algorithmic bias and the perpetuation of health inequities: an AI bias aware framework. Health Policy Technol. 2023;12:100702. 10.1016/j.hlpt.2022.100702

[19] Xu J , Xiao Y, Wang WH, et al Algorithmic fairness in computational medicine. EBioMedicine. 2022;84:104250. 10.1016/j.ebiom.2022.104250 36084616 PMC9463525

[20] Mccradden M , Odusi O, Joshi S, et al What’s fair is… fair? Presenting JustEFAB, an ethical framework for operationalizing medical ethics and social justice in the integration of clinical machine learning: JustEFAB. In: *2023 ACM Conference on Fairness, Accountability, and Transparency*. ACM; 2023:1505-1519. 10.1145/3593013.3594096

[21] Movva R , Shanmugam D, Hou K, et al Coarse race data conceals disparities in clinical risk score performance. *Proc Mach Learn Healthc Conf*. 2023;219:443-472. https://proceedings.mlr.press/v219/movva23a.html

[22] Meng C , Trinh L, Xu N, Enouen J, Liu Y. Interpretability and fairness evaluation of deep learning models on MIMIC-IV dataset. Sci Rep. 2022;12:7166. 10.1038/s41598-022-11012-2 35504931 PMC9065125

[23] Mohammed S , Matos J, Doutreligne M, Celi LA, Struja T. Racial disparities in invasive ICU treatments among septic patients: high resolution electronic health records analysis from MIMIC-IV. Yale J Biol Med. 2023;96:293-312. 10.59249/WDJI8829 37780990 PMC10524813

[24] Johnson AEW , Bulgarelli L, Shen L, et al MIMIC-IV, a freely accessible electronic health record dataset. Sci Data. 2023;10:1. 10.1038/s41597-022-01899-x 36596836 PMC9810617

[25] Glorot X , Bengio Y. Understanding the difficulty of training deep feedforward neural networks. In: Proceedings of the Thirteenth International Conference on Artificial Intelligence and Statistics, Vol. 9. 2010:249–256.

[26] Kouhounestani M , Song L, Luo L, Aickelin U. Enhancing predictive modeling in emergency departments. In: *Proceedings of the 10th International Conference on Information and Communication Technologies for Ageing Well and e-Health*. SCITEPRESS—Science and Technology Publications; 2024:37-46. 10.5220/0012568900003699

[27] Feretzakis G , Sakagianni A, Anastasiou A, et al Machine learning in medical triage: a predictive model for emergency department disposition. Appl. Sci. 2024;14:6623. 10.3390/app14156623

[28] Escobedo LE , Cervantes L, Havranek E. Barriers in healthcare for Latinx patients with limited English proficiency—a narrative review. J Gen Intern Med. 2023;38:1264-1271. 10.1007/s11606-022-07995-3 36720766 PMC9888733

[29] Fields A , Abraham M, Gaughan J, Haines C, Hoehn KS. Language matters: race, trust, and outcomes in the pediatric emergency department. Pediatr Emerg Care. 2016;32:222-226. 10.1097/PEC.0000000000000453 27031004

[30] Agarwal AK , Sagan C, Gonzales R, et al Assessing experiences of racism among Black and White patients in the emergency department. J Am Coll Emerg Physicians Open. 2022;3:e12870. h 10.1002/emp2.12870 PMC9772489 36570372

[31] Kang H , Zhang P, Lee S, Shen S, Dunham E. Racial disparities in opioid administration and prescribing in the emergency department for pain. Am J Emerg Med. 2022;55:167-173. 10.1016/j.ajem.2022.02.043 35358938

[32] Gil LA , Asti L, Beyene TJ, Cooper JN, Minneci PC, Besner GE. Inequities in the diagnosis of pediatric appendicitis in tertiary children’s hospitals and the consequences of delayed diagnosis. J Surg Res. 2023;292:158-166. 10.1016/j.jss.2023.07.049 37619501

[33] Braveman PA , Cubbin C, Egerter S, Williams DR, Pamuk E. Socioeconomic disparities in health in the United States: what the patterns tell us. Am J Public Health. 2010;1001:S186-S196. 10.2105/AJPH.2009.166082 PMC2837459 20147693

[34] White HL , O’Campo P, Moineddin R, Matheson FI. Modeling the cumulative effects of social exposures on health: moving beyond disease-specific models. Int J Environ Res Public Health. 2013;10:1186-1201. 10.3390/ijerph10041186 23528813 PMC3709312

[35] Davidson T , Mirza F, Baig MM. Impact of socio-economic status and race on emergency hospital admission outcomes: analysis from hospital admissions between 2001 and 2012. Health Serv Manage Res. 2022;35:127-133. 10.1177/09514848211012189 34107791

[36] Agarwal AK , Gonzales RE, Sagan C, et al Perspectives of Black patients on racism within emergency care. JAMA Health Forum. 2024;5:e240046. 10.1001/jamahealthforum.2024.0046 38457129 PMC10924244

[37] Mittelstadt BD , Allo P, Taddeo M, Wachter S, Floridi L. The ethics of algorithms: mapping the debate. Big Data Soc. 2016;3:2053951716679679. 10.1177/2053951716679679

[38] Kostick-Quenet KM , Cohen IG, Gerke S, et al Mitigating racial bias in machine learning. J Law Med Ethics. 2022;50:92-100. 10.1017/jme.2022.13 35243993 PMC12140104

[39] Jindal A. Misguided artificial intelligence: how racial bias is built into clinical models. J Brown Hosp Med. 2022;2:38021. 10.56305/001c.38021 PMC11878858 40046549

[40] Lett E , Shahbandegan S, Barak-Corren Y, Fine AM, La Cava WG. Intersectional and marginal debiasing in prediction models for emergency admissions. JAMA Netw Open. 2025;8:e2512947. 10.1001/jamanetworkopen.2025.12947 40440013 PMC12123471

[41] Zhang X , Wang H, Yu G, Zhang W. Machine learning-driven prediction of hospital admissions using gradient boosting and GPT-2. Digit Health. 2025;11:20552076251331319. 10.1177/20552076251331319 40162175 PMC11951900

[42] Hong WS , Haimovich AD, Taylor RA. Predicting hospital admission at emergency department triage using machine learning. PLoS One. 2018;13:e0201016. 10.1371/journal.pone.0201016 30028888 PMC6054406

[43] Hernandez-Boussard T , Bozkurt S, Ioannidis JPA, Shah NH. MINIMAR (minimum information for medical AI reporting): developing reporting standards for artificial intelligence in health care. J Am Med Inform Assoc. 2020;27:2011-2015. 10.1093/jamia/ocaa088 32594179 PMC7727333

[44] Moons KGM , Damen JAA, Kaul T, et al PROBAST + AI: an updated quality, risk of bias, and applicability assessment tool for prediction models using regression or artificial intelligence methods. BMJ. 2025;388:e082505. 10.1136/bmj-2024-082505 40127903 PMC11931409

