# Supplementary material for: Assessing the effect of race on machine learning models predicting hospital admissions from emergency department
Source: JAMIA Open. 2026 Aug 10;9(4):ooag157. doi: 10.1093/jamiaopen/ooag157 (PMC13457077; doi:10.1093/jamiaopen/ooag157)
Supplement: Supplementary file 1 [file ooag157_supplementary_data.docx]

**SUPPLEMENTAL TABLES**

| **Variable** | **Distribution** | **Preprocessing** | **Final Representation** |
| --- | --- | --- | --- |
| Time of day | 14.42 [10.58, 18.47]; 13.97 ± 5.84 | Extracted from arrival time as hour plus minutes/60; min–max scaled to [0, 1] | 1 scalar |
| Temperature | 98.0 [97.6, 98.6];  98.10 ± 0.92 | Kept if non-missing and within 96–104; min–max scaled to [0, 1] | 1 scalar |
| Heart rate | 84 [72, 96];  84.92 ± 17.36 | Kept if non-missing and within 20–300; min–max scaled to [0, 1] | 1 scalar |
| Respiratory rate | 18 [16, 18];  17.51 ± 2.22 | Kept if non-missing and within 5–80; min–max scaled to [0, 1] | 1 scalar |
| Oxygen saturation | 99 [97, 100];  98.42 ± 1.96 | Kept if non-missing and within 70–100; min–max scaled to [0, 1] | 1 scalar |
| Systolic blood pressure | 133 [120, 148];  135.12 ± 22.05 | Kept if non-missing and within 50–250; min–max scaled to [0, 1] | 1 scalar |
| Diastolic blood pressure | 77 [68, 87];  77.49 ± 14.49 | Kept if non-missing and within 30–150; min–max scaled to [0, 1] | 1 scalar |
| Triage acuity | 3 [2, 3];  2.67 ± 0.67 | Kept if non-missing and within 1–5; min–max scaled to [0, 1] | 1 scalar |
| Age | 53 [34, 68];  52.66 ± 20.56 | Min–max scaled to [0, 1] | 1 scalar |
| Gender | F 54.8%; M 45.2% | One-hot encoded (F, M) | 2 binary indicators |
| Arrival transport | WALK IN 63.8%;  AMBULANCE 36.2% | Restricted to WALK IN and AMBULANCE; one-hot encoded | 2 binary indicators |
| Race | WHITE 58.5%;  BLACK 22.4%;  HISPANIC 8.3%;  ASIAN 4.5%;  OTHER 6.3% | Race categories grouped; one-hot encoded | 5 binary indicators  (when race is included) |
| Chief complaint | Lowercased text;  top 5 full-text complaints: abd pain (14,737), chest pain (12,508), s/p fall (6,515), dyspnea (6,349), wound eval (4,740) | Lowercased; TF-IDF encoding (min_df = 10), fit on training set only | 1,018 TF-IDF features |

**Supplemental Table 1** – Distribution and Preprocessing of Model Input Variables. For scalar variables, the median [IQR] and mean ± standard deviation are reported. For binary indicators, proportions (%) are reported.

| **Unique Entries** | |
| --- | --- |
| **1** | AMERICAN INDIAN/ALASKA NATIVE |
| **2** | ASIAN |
| **3** | ASIAN - ASIAN INDIAN |
| **4** | ASIAN - CHINESE |
| **5** | ASIAN - KOREAN |
| **6** | ASIAN - SOUTH EAST ASIAN |
| **7** | BLACK/AFRICAN |
| **8** | BLACK/AFRICAN AMERICAN |
| **9** | BLACK/CAPE VERDEAN |
| **10** | BLACK/CARIBBEAN ISLAND |
| **11** | HISPANIC OR LATINO |
| **12** | HISPANIC/LATINO - CENTRAL AMERICAN |
| **13** | HISPANIC/LATINO - COLUMBIAN |
| **14** | HISPANIC/LATINO - CUBAN |
| **15** | HISPANIC/LATINO - DOMINICAN |
| **16** | HISPANIC/LATINO - GUATEMALAN |
| **17** | HISPANIC/LATINO - HONDURAN |
| **18** | HISPANIC/LATINO - MEXICAN |
| **19** | HISPANIC/LATINO - PUERTO RICAN |
| **20** | HISPANIC/LATINO - SALVADORAN |
| **21** | MULTIPLE RACE/ETHNICITY |
| **22** | NATIVE HAWAIIAN OR OTHER PACIFIC ISLANDER |
| **23** | OTHER |
| **24** | PATIENT DECLINED TO ANSWER |
| **25** | PORTUGUESE |
| **26** | SOUTH AMERICAN |
| **27** | UNABLE TO OBTAIN |
| **28** | UNKNOWN |
| **29** | WHITE |
| **30** | WHITE - BRAZILIAN |
| **31** | WHITE - EASTERN EUROPEAN |
| **32** | WHITE - OTHER EUROPEAN |
| **33** | WHITE - RUSSIAN |

**Supplemental Table 2** – Unique Race and Ethnicity Entries in the MIMIC-IV Dataset.

|  | **Effect size** | | |
| --- | --- | --- | --- |
|  | **Baseline** | **Balanced** | **Randomized Balanced** |
| **White** | **1.00** | **1.22** | 0.13 |
| **Black** | **-1.23** | -0.65 | 0.22 |
| **Hispanic** | **-1.35** | **-1.00** | -0.03 |
| **Other** | -0.63 | 0.14 | 0.52 |
| **Asian** | -0.67 | 0.23 | -0.18 |

**Supplemental Table 3** – Effect size for different racial groups across the baseline, balanced, and randomized-balanced validation datasets. Large effect sizes are bolded.

**SUPPLEMENTAL FIGURES**

**Supplemental Figure 1** – Distribution of Original Race Categories After Preliminary Preprocessing the MIMIC-IV Dataset. The preliminary preprocessing entails grouping race categories based on the first word of the original descriptor (e.g., “WHITE - xxxx” → “White”).

**Supplemental Figure 2** – Validation Set Admission Rates by Race and Training Set Exposure.

**Supplemental Figure 3** – Distribution of Race in the Unfiltered Dataset Prior to Exclusion for Missingness. The distribution of race in the unfiltered dataset prior to exclusion for missing or out-of-range values is shown. Compared to Supplemental Figure 1 (post-processing), the overall distribution remains largely unchanged, suggesting that data filtering did not substantially alter the racial composition of the cohort.
